# Reduced Coupling of Oxidative Phosphorylation *In Vivo* Precedes Electron Transport Chain Defects Due to Mild Oxidative Stress in Mice

**DOI:** 10.1371/journal.pone.0026963

**Published:** 2011-11-22

**Authors:** Michael P. Siegel, Shane E. Kruse, Gary Knowels, Adam Salmon, Richard Beyer, Hui Xie, Holly Van Remmen, Steven R. Smith, David J. Marcinek

**Affiliations:** 1 Department of Bioengineering, University of Washington Medical School, Seattle, Washington, United States of America; 2 Department of Radiology, University of Washington Medical School, Seattle, Washington, United States of America; 3 Department of Environmental and Occupational Health Sciences, University of Washington Medical School, Seattle, Washington, United States of America; 4 Translational Research Institute for Metabolism and Diabetes, Florida Hospital, Sanford-Burnham Medical Research Institute, Winter Park, Florida, United States of America; 5 Department of Cellular and Structural Biology, University of Texas Health Sciences Center, San Antonio, Texas, United States of America; University of Valencia, Spain

## Abstract

Oxidative stress and mitochondrial function are at the core of many degenerative conditions. However, the interaction between oxidative stress and *in vivo* mitochondrial function is unclear. We used both pharmacological (2 week paraquat (PQ) treatment of wild type mice) and transgenic (mice lacking Cu, Zn-superoxide dismutase (SOD1^−/−^)) models to test the effect of oxidative stress on *in vivo* mitochondrial function in skeletal muscle. Magnetic resonance and optical spectroscopy were used to measure mitochondrial ATP and oxygen fluxes and cell energetic state. In both models of oxidative stress, coupling of oxidative phosphorylation was significantly lower (lower P/O) at rest *in vivo* in skeletal muscle and was dose-dependent in the PQ model. Despite this reduction in efficiency, *in vivo* mitochondrial phosphorylation capacity (ATPmax) was maintained in both models, and *ex vivo* mitochondrial respiration in permeabilized muscle fibers was unchanged following PQ treatment. In association with the reduced P/O, PQ treatment led to a dose-dependent reduction in PCr/ATP ratio and increased phosphorylation of AMPK. These results indicate that oxidative stress uncouples oxidative phosphorylation *in vivo* and results in energetic stress in the absence of defects in the mitochondrial electron transport chain.

## Introduction

Oxidative stress and mitochondrial function are key elements of many pathological conditions, including high fat diet induced insulin resistance, neurodegenerative disease, disuse atrophy, and sarcopenia [1], [2], [3], [4], [5]. The role of reactive oxygen species (ROS) in cellular function is most commonly associated with damage to DNA, proteins, and lipids. However, ROS are also involved in the regulation of several cellular processes including mitochondrial biogenesis [6], [7], increased activation of antioxidant defenses [8], [9], [10], exercise adaptation in skeletal muscle [6], [11], [12], [13] and cell death [14], [15]. Mitochondrial function and energy homeostasis play a key role in many of these processes. Thus, interaction between mitochondrial function and oxidative stress appears to play an important role in controlling cell health and disease [14], [16], [17], [18]. However, the control of *in vivo* mitochondrial function and cell energetics by oxidative stress remains unclear.

In skeletal muscle, ROS are produced by the mitochondrial electron transport chain as a by-product of oxygen consumption [19] and by cytosolic sources such as NAD(P)H oxidase during contraction [12]. ROS or oxidative by-products modulate mitochondrial metabolism *in vitro* beyond the mitochondrial dysfunction that results from oxidative damage. Addition of oxidants or lipid peroxides to isolated mitochondria results in an increased proton leak from the inner membrane space into the matrix thereby partially uncoupling mitochondrial ATP production from oxygen consumption [20], [21]. Rather than being caused by damage to mitochondrial proteins or lipids, this reduced coupling is the result of activated proton leak proposed to be mediated by uncoupling protein 3 (UCP3) and adenine nucleotide translocase (ANT) [22]. However, activated leak through UCP3 and ANT is enhanced in the presence of fatty acids and is inhibited by physiological concentrations of GDP and other purine nucleotides *in vitro* [22], [23]. Thus, it is not clear to what extent this oxidative stress-activated leak occurs *in vivo* and, if present, what effect it has on cell energetics.

In this study we use metabolic spectroscopy to test the control of *in vivo* mitochondrial coupling by oxidative stress. We use magnetic resonance (MR) spectroscopy and optical spectroscopy [24] to determine the coupling of oxidative phosphorylation (P/O), maximal mitochondrial ATP production (ATPmax), and cell energy state in mice treated with paraquat (PQ) and mice lacking the antioxidant enzyme Cu-Zn-superoxide dismutase (SOD1^−/−^). We find that mild oxidative stress results in reduced P/O and energy stress *in vivo* in mouse skeletal muscle before the development of intrinsic mitochondrial defects.

## Results

### PQ treatment leads to oxidative stress in skeletal muscle

**Figure 1:**
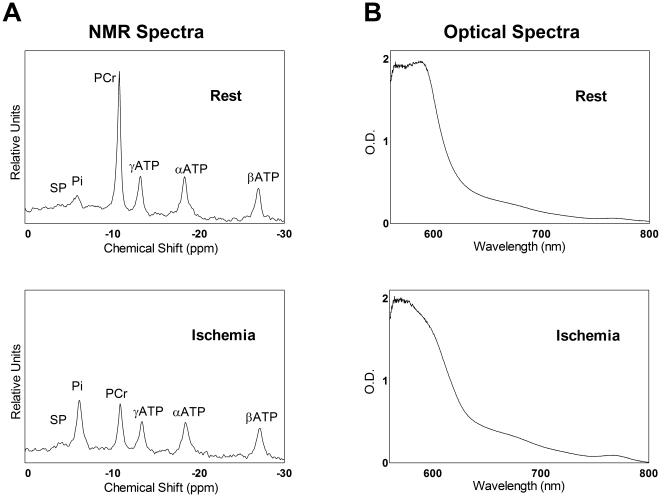
*In vivo* spectra exhibit distinct differences under resting and ischemic conditions. (A) MR spectra collected during rest (top) and after 10 min of ischemia (bottom). (B) Optical spectra collected at rest (top) and after 6 min of ischemia (bottom).

We used MR and optical spectroscopy to measure the effect of increased oxidative stress on *in vivo* mitochondrial function in skeletal muscle. Representative ^31^P MR and optical spectra of the mouse hindlimb during rest and at the end of ischemia are illustrated in Fig. 1. MR spectra of skeletal muscle reveal the decrease in phosphocreatine (PCr) during ischemia while ATP levels remain constant (Fig. 1A). Optical spectra become broader with a more pronounced peak at 760 nm as hemoglobin and myoglobin transition from their oxygenated to deoxygenated states during ischemia (Fig. 1B). Table 1 lists resting levels for key metabolites used to determine *in vivo* mitochondrial fluxes.

**Table 1:** Metabolite concentrations in muscles of the mouse hindlimb.

Study	Group	PCr/ATP	ATP(mM)	Cr (mM)	PCr (mM)	Pi (mM)	pH_rest_
**PQ**	C	3.77±0.04	9.52±0.41	49.2±1.5	35.77±0.36	2.52±0.23	7.11±0.03
	PQ1	3.62±0.06	10.46±0.28	51.9±2.3	*38.02±0.65* *	2.99±0.31	7.07±0.02
	PQ2	*3.53±0.09* *	9.82±0.20	49.3±1.4	34.56±0.88	3.04±0.23	7.13±0.02
**SOD1**	WT	3.61±0.16	8.00±0.92	41.1±2.6	28.90±2.56	3.11±0.17	7.08±0.02
	SOD1^−/−^	3.84±0.13	*6.48±0.80* *	*30.0±3.6* *	*25.50±1.61* *	3.77±0.34	7.14±0.03

ATP and total creatine (Cr) were determined using HPLC, and other values were calculated using MR spectra with ATP as an internal standard. All data are expressed as mean ± SEM. n = 8–9 for PQ and n = 4–5 for SOD1.

* p<0.05 relative to control.

**Figure 2:**
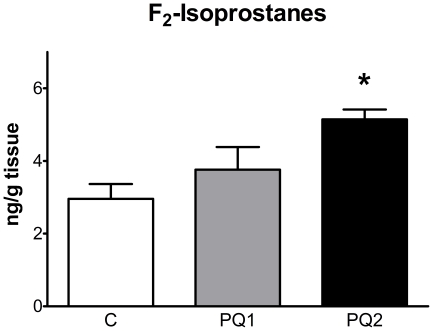
PQ-treatment caused a dose-dependent increase in oxidative stress in mouse skeletal muscle. F_2_-isoprostanes increased in skeletal muscle with PQ treatment. Data are expressed as means ± SEM with n = 4–5 per group. (* p<0.05 and ** p<0.01 relative to control).

To better understand the effect of short term oxidative stress we treated wild-type C57Bl/6J mice with low doses of PQ to induce a mild oxidative stress. Mice were injected either once (PQ1) or twice (PQ2) a week for two weeks with 10 mg PQ/kg body weight, while controls (C) received volume-matched doses of saline. We measured F_2_-isoprostanes in skeletal muscle of the hindlimb as a marker of oxidative stress. Figure 2 shows that PQ treatment induced a significant, dose-dependent oxidative stress in skeletal muscle. We also observed significant differences in body weight in both PQ1 (0.3%±1.2% decrease, p<0.05) and PQ2 (2.9%±1.2% decrease, p<0.01) over the course of treatment relative to controls (5.8%±2.6% increase). To ensure that our measurements of *in vivo* mitochondrial function were not affected by systemic effects of PQ treatment on the cardiovascular system we measured resting heart rate and arterial oxygen saturation in anesthetized mice (Methods S1) and found no effect of PQ treatment on these parameters (Fig. S1).

### Reduced P/O and energy stress with PQ treatment

**Figure 3:**
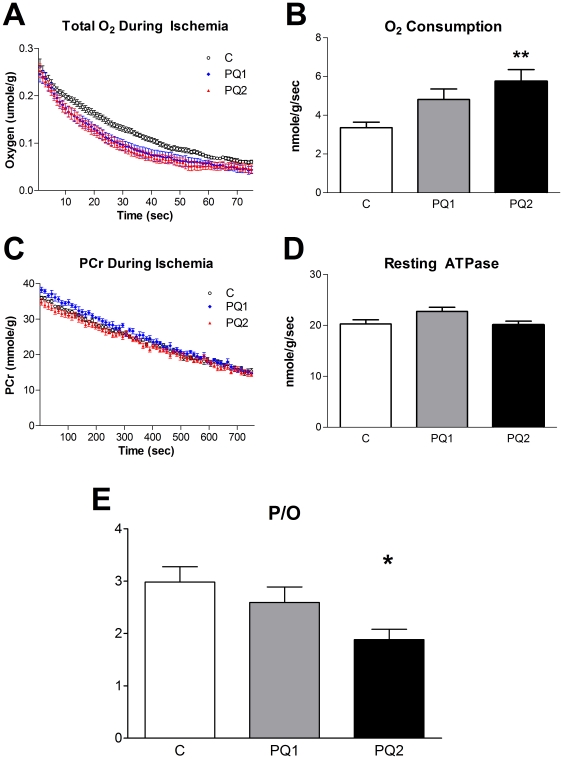
The effects of PQ treatment on *in vivo* mitochondrial metabolism. PQ led to a significant, dose-dependent increase in oxygen consumption (A and B; pre-oxygen limited slope of A used to calculate values represented in B) but had only a minor effect on resting ATP production (ATPase) rate (C and D; slope of C used to calculate values represented in D). The ratio of ATP produced per O_2_ consumed (P/O ratio) is significantly decreased in a dose-dependent manner with PQ treatment (E). Data are expressed as means ± SEM with n = 8–9 per group. (* p<0.05, ** p<0.01 relative to control).

Resting *in vivo* mitochondrial oxygen consumption in the hindlimb skeletal muscle increased significantly with PQ treatment (Fig. 3A and 3B). In addition, resting *in vivo* myoglobin saturation was negatively correlated with oxygen consumption due to the increased oxygen demand of the muscle (Fig. S2). However, the resting rate of mitochondrial ATP production was unchanged (Fig. 3C and 3D). This resulted in a dose-dependent decrease in P/O ratio (ATP production/O_2_ consumption divided by 2) with PQ treatment (Figs. 3E and S3A).

**Figure 4:**
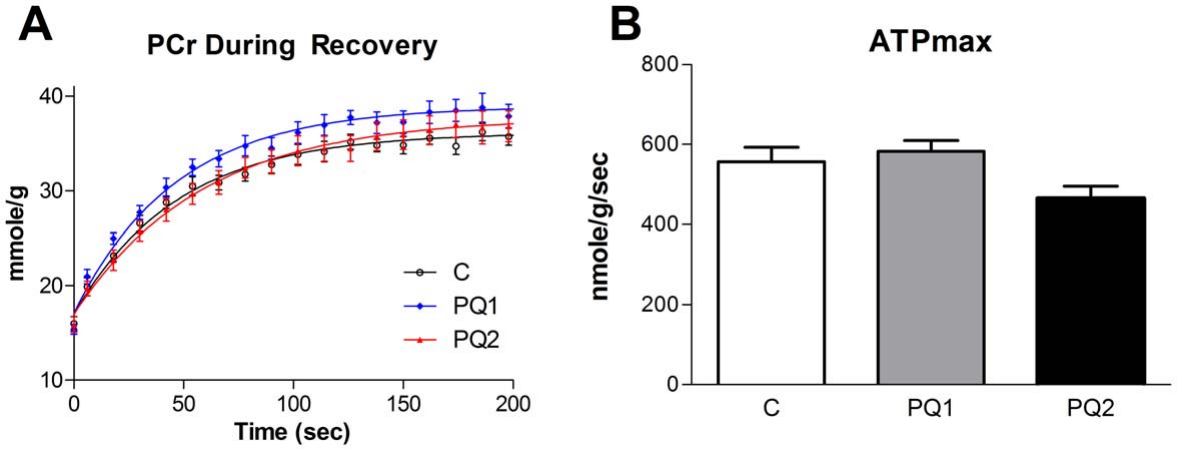
Mild oxidative stress does not effect *in vivo* ATPmax. Neither PCr recovery (A) nor ATPmax (B) changed significantly with either dose of PQ, although the decrease in ATPmax of group PQ2 trended down (p<0.1). Data are expressed as means ± SEM with n = 8–9 per group. (* p<0.05 relative to control).

To measure the effect of increased oxidative stress on the functional capacity of mitochondria we determined *in vivo* ATPmax by measuring the rate of recovery of PCr from ischemia in the mouse hindlimb [25]. Despite the reduction in P/O ratio, ATPmax was not different between groups (Fig. 4).

**Figure 5:**
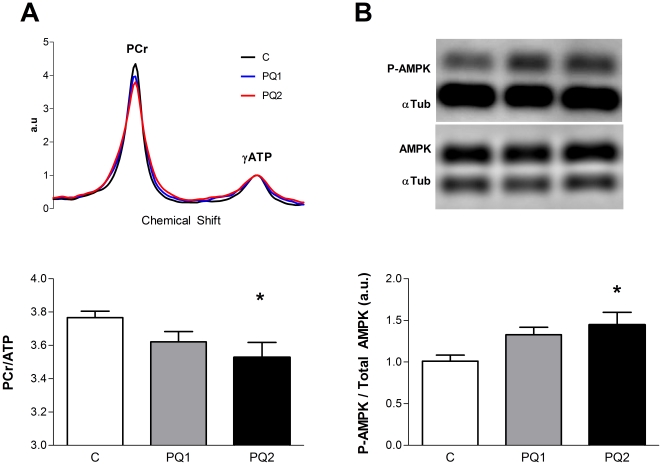
PQ-treatment causes energy stress *in vivo* and activates energy sensing pathways. PCr/ATP ratio decreases with PQ treatment (A, with representative MR spectra above) and the ratio of phosphorylated AMPK to total AMPK increased (B, with representative western blot above). Data are expressed as means ± SEM with n = 5–9 per group. AMPK data are normalized to control average. (* p<0.05 relative to control).

Biochemical and molecular evidence indicates that PQ treatment disrupted energy homeostasis *in vivo* in skeletal muscle. The PCr/ATP ratio in resting skeletal muscle declined in a dose-dependent manner with increasing PQ treatment (Fig. 5A and S3B). This decrease in PCr/ATP led to a trend toward elevated AMP concentrations in both treated groups (C: 104.4 nM±16.1 nM, PQ1: 156.7 nM±48.0 nM, PQ2: 174.5 nM±38.3 nM). Further evidence that PQ treatment led to a disruption in energy homeostasis comes from increased phosphorylation of the cell energy sensing protein, 5′ AMP-activated protein kinase (AMPK) in the PQ2 group relative to controls (Fig. 5B).

### Mild PQ treatment does not affect intrinsic mitochondrial function

**Figure 6:**
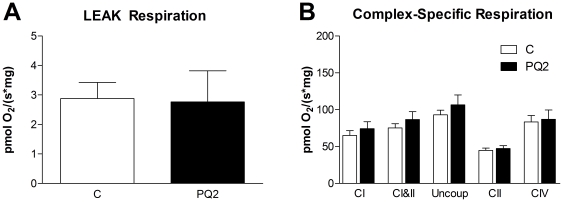
PQ-treatment had no effect on *ex vivo* respiration of permeabilized EDL. (A) We detected no difference in leak driven respiration between control and PQ2 in the permeabilized EDL. (B) State 3 with complex I substrate (“CI”), state 3 with complex I and II substrates (“CI&II”), uncoupled (“Uncoup”), uncoupled with complex I inhibition (“CII”), and complex IV (“CIV”) respiration were also unchanged in group PQ2. Data are expressed as means ± SEM with n = 5 per group.

To determine whether the reduction in *in vivo* P/O with PQ treatment was due to changes intrinsic to the mitochondria or due to interactions between mitochondria and the cellular environment (e.g. ROS) we measured mitochondrial respiration in permeabilized muscle fibers following *in vivo* studies. We found no effect of PQ treatment on proton leak driven respiration, maximal ADP stimulated respiration with complex I substrate (state 3, complex I), maximal ADP stimulated respiration with complex I and II substrates (state 3, complex I & II), uncoupled respiration, uncoupled respiration with complex I inhibition (i.e. complex II only), or complex IV respiration (Figs. 6A and 6B). These results indicate that mitochondrial electron transport chain function was not affected by this mild oxidative stress.

**Figure 7:**
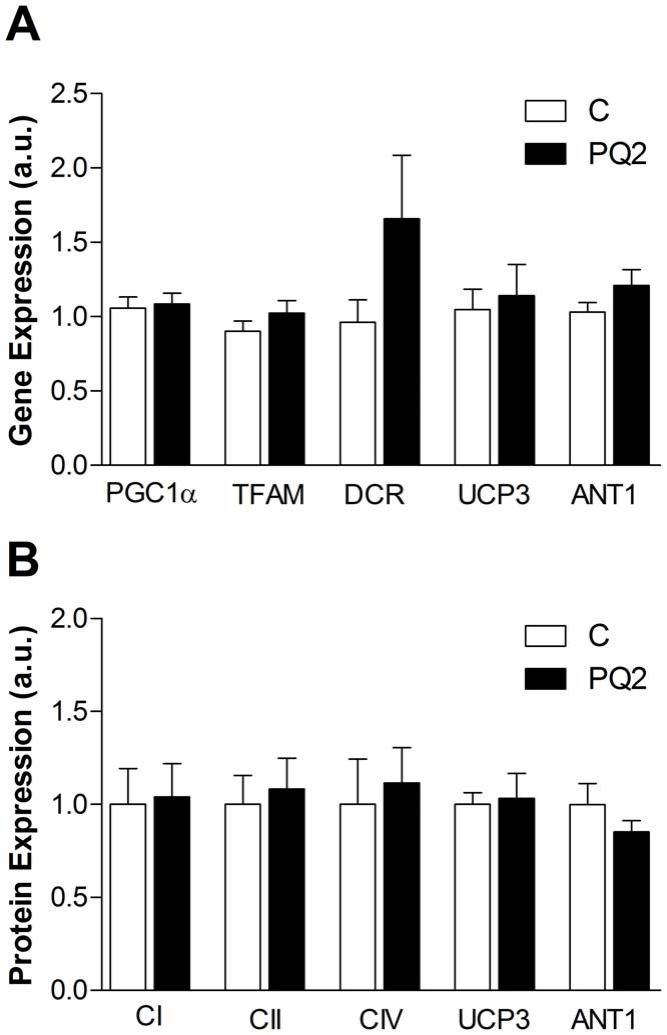
PQ treatment did not alter mitochondrial content or composition. (A) We detected no differences between control and PQ2 in gene expression of mitochondrial regulators PGC1α and TFAM, the d-loop control region of mitochondrial DNA (DCR), UCP3, or ANT1. (B) We also did not detect changes in protein expression of complexes I, II, or IV, UCP3, or ANT1 in PQ2 animals. Data are expressed as means ± SEM, normalized to control averages. n = 5 per group.

We next looked for changes in expression of genes and proteins relevant to mitochondrial content and function in mixed gastrocnemius to account for loss of *in vivo* P/O and maintenance of mitochondrial capacity. We found no significant effect of PQ treatment on gene expression of peroxisome proliferator-activated receptor gamma coactivator 1- α (PGC1α), mitochondrial transcription factor A (TFAM), the D-loop control region of mitochondrial DNA (DCR), UCP3, or ANT1 (Fig. 7A). There was also no difference in the content of the mitochondrial proteins NDUFB8 of complex I, 30 kDa subunit of complex II, subunit IV of cytochrome oxidase, UCP3, or ANT1 (Fig. 7B).

### Oxidative stress leads to *in vivo* mitochondrial uncoupling in SOD1^−/−^ mice

**Figure 8:**
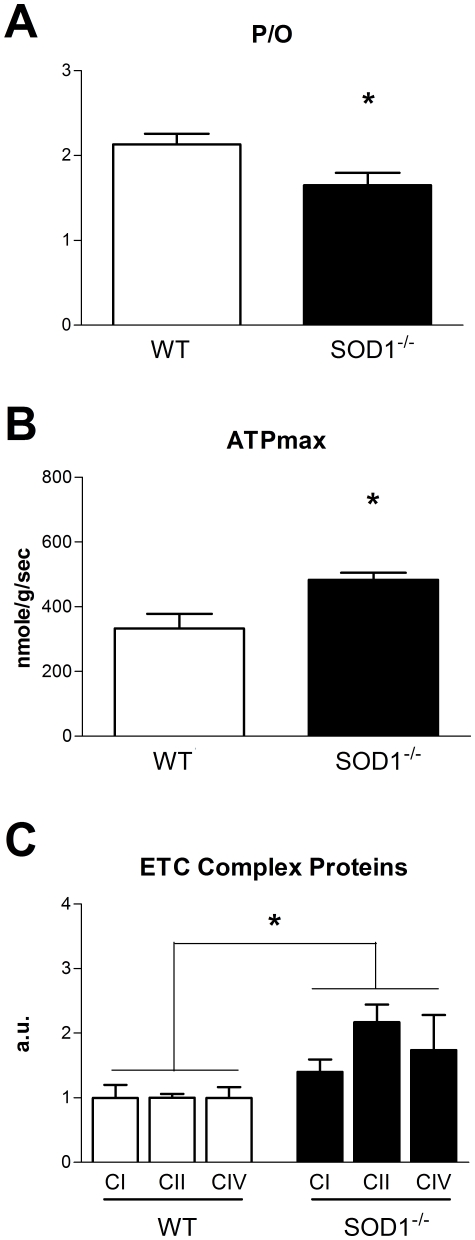
*In vivo* mitochondrial metabolism in SOD1^−/−^ mice. (A) Coupling of oxidative phosphorylation was lower in SOD1^−/−^ mice, as reflected by a significant decrease in P/O. (B) Despite this loss of efficiency, ATPmax was higher in SOD1^−/−^ mice. (C) Protein expression of complexes I, II, and IV was also significantly higher in SOD1^−/−^ mice. Data are expressed as means ± SEM with n = 4–5 per group. In C, data are normalized to control averages. Significant effect of genotype determined by two-way ANOVA. (* p<0.05).

As reported previously, the absence of SOD1 leads to increased oxidative stress in mice [5]. We confirmed this finding as an increase in F_2_-isoprostanes in skeletal muscle of SOD1^−/−^ mice (Fig. S4). While neither *in vivo* mitochondrial ATP production nor oxygen consumption was significantly different in SOD1^−/−^ mice (data not shown), P/O ratio was significantly reduced (Fig. 8A). In addition, resting ATP and total creatine concentrations were lower in SOD1^−/−^ mice compared to wild-type (WT) littermates (Table 1).

### Increased phosphorylation capacity in SOD1^−/−^ skeletal muscle

We found that ATPmax was significantly higher in mice lacking SOD1 (Fig. 8B). This increase in ATPmax contrasts with the effect of short term oxidative stress in PQ-treated mice and suggests a compensatory increase in mitochondrial content with chronic oxidative stress in SOD1^−/−^ mice.

Elevation of electron transport chain complexes I, II, and IV in the mixed gastrocnemius of SOD1^−/−^ mice (Fig. 8C) was associated with the increased ATPmax. DNA microarray analysis of gene expression was used to further examine the mitochondrial response to the absence of SOD1 (Methods S1, Tables S1 and S2). Using NIH DAVID to examine the list of genes that were changed in the extensor digitorum longus (EDL) of SOD1^−/−^ mice compared to WT we found that many genes involved in metabolic processes or mitochondria were significantly upregulated, while genes involved in the nuclear compartment and transcriptional and translational processing were significantly downregulated (Fig. S5).

## Discussion

We demonstrate that mild oxidative stress induced by either PQ treatment or SOD1 knockout reduces the coupling of oxidative phosphorylation *in vivo* and disrupts skeletal muscle energetics. These results provide an *in vivo* test of the “uncoupling to survive” hypothesis, which proposes that oxidative stress activates mitochondrial proton leak and reduces the coupling of oxidative phosphorylation [26]. Production of both ATP and ROS are linked to the inner mitochondrial membrane potential (Δψ), which is generated as protons are pumped from the matrix to the inner membrane space by the oxygen-consuming redox reactions of the electron transport chain [27], [28]. The “uncoupling to survive” hypothesis suggests that oxidative stress leads to an increase in mitochondrial proton leak, and therefore a reduction of Δψ, as a negative-feedback mechanism that mitigates further production of mitochondrial ROS [19]. As a result of this proton leak, the F_1_F_0_ATPsynthase is bypassed and the net result is a reduction in the ATP produced per oxygen consumed (lower P/O).

*In vivo* P/O values were significantly reduced in both short-term (2 week PQ treatment) and chronic (SOD1^−/−^) models. The 10 mg/kg of PQ administered once or twice per week in this study was sufficient to induce an increase in markers of oxidative stress, but this PQ dose is well below the LD50 of 70 mg/kg reported for mice when PQ is dissolved in saline and administered intraperitoneally [29]. Thus the absence of an acute effect on oxygen delivery to skeletal muscle or severe mitochondrial dysfunction is not surprising. Instead we observed a significant control of mitochondrial metabolism and energetics. Resting mitochondrial ATP production rate fluctuated less than 15% with PQ treatment indicating that this level of oxidative stress did not greatly alter resting ATP demand of skeletal muscle. However, oxygen consumption increased by 43% in PQ1 and 71% in PQ2 to meet this unchanged ATP demand as a result of the reduced coupling of oxidation to phosphorylation. These results are similar to those from isolated mitochondria and cells, where increased proton leak can be induced by adding exogenous oxidants to the assay [21], [30].

We used permeabilized muscle fibers to test whether the *in vivo* effects on mitochondrial function were due to changes intrinsic to the mitochondria or were the result of the interaction between the mitochondria and the cellular environment. No difference in mitochondrial respiration in permeabilized EDL between the PQ-treated and control groups, including proton leak driven respiration, indicates that the decreased coupling observed *in vivo* was not due to oxidative damage or a change in the composition of the mitochondria. These changes would be intrinsic to the mitochondria and persist after the fibers were permeabilized and the cell environment was replaced by respiration buffer. Further, analysis of gene and protein expression in mixed gastrocnemius revealed no differences in regulators of mitochondrial biogenesis (PGC1α and TFAM), mitochondrial content (DCR and complexes I, II, and IV), or mitochondrial uncouplers (UCP3 and ANT1) in PQ-treated mice. Therefore, we conclude that the reduced *in vivo* P/O is due to direct control of mitochondrial function by ROS or a related by-product.

Reduced *in vivo* coupling with PQ was not due to an upregulation of UCP3 or ANT1 expression in the muscle. The difference between our results and previous reports indicating that UCP3 and ANT1 are upregulated in response to oxidative stress may be due to differences in the magnitude of the oxidative stress or the short duration of the treatment. The previous studies in cell culture measured expression levels hours following an acute, high dose oxidative stress [8], while the PQ treatment employed in this study involved a relatively low level of stress induced over a period of two weeks. The lack of difference in expression levels of UCP3 and ANT1 does not preclude a role for these proteins in the reduced mitochondrial P/O *in vivo*. Activation of UCP3 or ANT1 mediated proton leak by oxidative stress or lipid peroxides [20], [21], [31] *in vivo* provides a potential mechanism to explain the difference between our *in vivo* and permeabilized fiber results. Exchange of the cell environment and loss of potential activators of UCP3 or ANT1 during the permeabilization process would reverse the increased proton leak measured *in vivo* with PQ treatment.

Chronic oxidative stress due to the absence of SOD1 also reduced the efficiency of oxidative phosphorylation. In previous studies, respiratory control ratios (state 3/state 4 respiration) in isolated mitochondria were significantly reduced in 20 month old SOD1^−/−^ mice, associated with decreased mitochondrial ATP production, loss of skeletal muscle mass, and increased mitochondrial H_2_O_2_ production [5], [32]. In this study, the elevated ATPmax and increased expression of mitochondrial proteins and transcripts in SOD1^−/−^ mice suggests that oxidative stress and uncoupling in young adult SOD1^−/−^ mice induce a compensatory increase in mitochondrial capacity. The more severe effects on mitochondrial dysfunction reported in SOD1^−/−^ mice at older ages are likely due to accumulation of oxidative damage to mitochondria and decline in ATP produced per mitochondria [32]. The increase in mitochondrial proteins in SOD1^−/−^ mice is supported by the growing evidence that ROS are important regulators of mitochondrial biogenesis. Treatment of renal proximal tubular cells and neural cells with H_2_O_2_ in culture induces mitochondrial biogenesis and upregulation of mitochondrial antioxidants [7], [8]. The absence of an effect of PQ treatment on mitochondrial biogenesis in this study may be due low level or short duration of the stress.

Reduced P/O is associated with disruption of energy homeostasis as indicated by the lower PCr/ATP ratios in PQ treated mice. The mitochondrial membrane potential provides a mechanistic link between cell energy state and the coupling of oxidative phosphorylation and imposes a thermodynamic limit on the ATP/ADP ratio that can be maintained [33]. Therefore, as resting membrane potential decreases due to the increased proton leak across the inner mitochondrial membrane, the resting ATP/ADP ratio also decreases leading to a lower PCr/ATP and elevated AMP levels through the creatine kinase and adenylate kinase reactions [34]. Thus we find that mild oxidative stress is associated with increased energy stress and elevated resting oxygen consumption in resting skeletal muscle.

Although PCr/ATP ratio was maintained in SOD1^−/−^ mice, the significant decrease in ATP concentration provides evidence of energy stress in the case of chronic oxidative stress. We have previously reported a loss of resting ATP levels *in vivo* associated with reduced P/O in aged skeletal muscle from both mouse [35] and humans [36]. However, we cannot rule out the possibility that lower ATP concentrations are due to heterogeneity of cell health and ATP concentration within the skeletal muscle of the hindlimb. Our *in vivo* MR and HPLC measurements of ATP are taken over the entire distal hindlimb, so we cannot account for local differences or loss of ATP in severely damaged fibers. These types of variation may reduce the volume averaged resting ATP levels and provide an alternative explanation for the lower ATP in SOD1^−/−^ mice.

Maintenance of cell energy homeostasis is an important determinant of cell health and survival [37]. Thus energy stress is a key adaptive signal that modifies gene and protein expression through multiple energy sensing signaling molecules such as AMPK and related kinases [38]. AMPK phosphorylation is sensitive to the cell ATP/AMP ratio [39], which is biochemically linked to the PCr/ATP ratio through the creatine kinase and adenylate kinase reactions [40], [41]. Activation of AMPK through increased phosphorylation controls downstream pathways to reduce energy use [42] and increase energy production [43] to restore energy homeostasis [39], [43]. However, increased activation of AMPK has also been shown to contribute to increases in cell death and muscle atrophy [44], [45], [46]. Therefore, an initial adaptive response by the cell to restore energy homeostasis may ultimately have pathological outcomes in the presence of a chronic energy or oxidative stress.

In conclusion, we demonstrate that mild oxidative stress leads to reduced mitochondrial coupling and cell energy stress *in vivo*. Thus reduced *in vivo* P/O may provide a mechanism that links mild oxidative stress to the activation of energy sensitive signaling processes. We demonstrate that this mitochondrial uncoupling and energy stress precedes severe defects in mitochondrial function or impaired ATP production and may serve as an early biomarker for disruption of normal mitochondrial function by oxidative stress.

## Methods

### Animals

This study was approved by the Institutional Animal Care and Use Committee of the University of Washington under protocol number 4130-01. Male and female wild type and SOD1^−/−^ [5] C57BL/6J mice between the ages of 4 and 8 months were housed in an environment with a fixed temperature, exposed to a 12 hour light/dark cycle, and allowed free access to standard mouse chow until immediately prior to experimentation. Mice were anesthetized using 5% (w/v) Avertin (1∶1 tribromoethanol in tert-amyl alcohol) in saline at a dose of 0.01 mL/gram of body weight. Body temperature was maintained at 35 +/− 1°C using heated air, and mice breathed 100% oxygen throughout experiments.

### Paraquat Treatment

Paraquat (Item 36541, Sigma, St. Louis, MO) was dissolved in saline and administered via intraperitoneal injections at a dose of 10 mg/kg of body weight. All mice received four injections over the span of 14 days (on days 1, 5, 8, and 12) for doses of 20 mg/kg/week (PQ2), 10 mg/kg/week (PQ1), or 0 mg/kg/week (C). Group PQ2 received 10 mg/kg paraquat on all four injection days. Group PQ1 received volume-matched doses of saline on days 1 and 8 and 10 mg/kg paraquat on days 5 and 12. Group C received volume-matched saline on all four injection days. *In vivo* spectroscopy commenced two days after the final injection in all cases.

### MR Spectroscopy

Detailed methods for MR spectroscopy are described elsewhere [24]. Briefly, anesthetized mice were secured horizontally in a vertical bore magnet (7 Tesla, Oxford Instruments, Oxford, UK) using flexible straps. The distal hindlimb was positioned within a three-turn solenoidal RF coil tuned to ^31^P (121.65 MHz), with a custom-built ischemia cuff positioned proximal to the coil. After shimming the proton peak using tissue water, a high signal to noise ^31^P spectrum was acquired under fully relaxed conditions (32 acquisitions with a 25 s interpulse delay) to calculate the peak areas of inorganic phosphate (P_i_), PCr, and ATP. We then collected 200 dynamic spectra (4 acquisitions per spectrum with a 1.5 s interpulse delay and a 45° flip angle for a time resolution of 6 s) during 2 min of rest, 11 min of ischemia, and 7 min of recovery to monitor the change in PCr peak area over time. Fully relaxed peak areas were calculated by integration of processed spectra using VNMR software on a Varian Inova console. For dynamic spectra, two consecutive spectra were summed to increase signal-to-noise (reducing time resolution to 12 s) and peak areas relative to a standard spectrum were determined using the Fit to Standard algorithm [47].

### Optical Spectroscopy

Detailed methods for optical spectroscopy are also available elsewhere [24]. After MR spectroscopy, mice were allowed to recover for 24 h and then re-anesthetized. Hindlimbs were shaved using standard hair clippers and mice were positioned on a flat horizontal surface with the distal portion of their hindlimbs oriented between two fiber optic bundles just downstream of an ischemia cuff. One bundle directed light from a quartz-tungsten halogen light source (Newport/Spectra-Physics, Santa Clara, CA) onto the hindlimb. Light transmitted through the hindlimb was acquired by the second bundle and delivered to a spectrograph (InSpectrum, Acton, Acton, MA). Optical spectra were acquired at 1 s intervals during 3 min of rest, 7 min of ischemia, and 10 min of recovery. Intensity spectra were converted to optical densities (OD) using a 1% intralipid reference. The second derivatives of OD spectra were analyzed using a partial least-squares (PLS) algorithm [48] to isolate the contributions to absorbance of oxymyoglobin, myoglobin, oxyhemoglobin, and hemoglobin.

### Ex Vivo Mitochondrial Respiration

Respiratory complex activities were measured by monitoring the rate of oxygen consumption in the presence of complex-specific substrates and inhibitors. Complex activity was measured when ATP production was coupled through a functional respiratory chain with oxygen as the final electron acceptor and also measured in the presence of the proton ionophore carbonylcyanide m-chlorophenylhydrazone (CCCP), providing rates of maximal electron flux independent of the phosphorylation systems. Freshly dissected, gently separated and permeabilized (50 µg/ml saponin, 4°C, 40 min) EDL muscle fibers were stirred at 25°C in a 2 ml vessel of an oxygen monitoring apparatus (O2k system, Oroboros Instruments, Austria) and provided with substrates in this order: for measuring proton leak (10 mM glutamate/5 mM pyruvate/2 mM malate, without ADP), state 3 with complex I substrate (previous conditions with 2.5 mM ADP), state 3 with complex I and II substrate (previous conditions with 10 mM succinate), uncoupled (previous conditions with 2.5 µM CCCP to measure maximal flux), uncoupled with complex I inhibition (i.e. complex II only; previous conditions with 0.5 µM rotenone), finally 2.5 µM antimycin A was added to measure the contribution of non-mitochondrial oxygen consumption followed by measurement of complex IV (0.5 mM N,N,N′,N′-tetramethyl-ρ-phenylenediamine (TMPD), 2 mM ascorbate). Potassium cyanide (1 mM) was used to confirm complex IV-specific activity. The amount of oxygen consumed was calculated by assuming the oxygen solubility in media to be 0.920 and by calibrating initial oxygen concentration in the buffer for each experiment and correcting for pressure, temperature, and instrumental oxygen consumption as described by Gnaiger et al. [49].

### Tissue Preparation

Muscles from the distal hindlimb (gastrocnemius, soleus, EDL, tibialis anterior) were dissected and flash-frozen in liquid nitrogen prior to death. These frozen muscles were pulverized over liquid nitrogen and mixed well to form a homogenous powder for F_2_-isoprostane, metabolite, hemoglobin, and myoglobin analysis. The gastrocnemius from the contralateral leg was dissected, frozen, and pulverized in liquid nitrogen for other biochemical analyses.

### Metabolite Concentration

Tissue concentrations of ATP and creatine were measured in mixed muscle preparations including gastrocnemius and tibialis muscle groups using HPLC (Waters, Milford, MA) following a previously published protocol [50]. We combined the gastrocnemius and tibialis muscle groups because they represent the bulk of the muscles measured in our *in vivo* experiments.

### Protein Content

Ten to 20 mg of pulverized muscle (whole leg or gastrocnemius) was homogenized in Cellytic MT (Item C3228, Sigma) with 0.015% (v/v) protease inhibitor (P8340, Sigma) and 1% (v/v) phosphatase inhibitor (Item #78420, Thermo Scientific, Waltham, MA) at a ratio of 1∶25 (w/v). Homogenates were combined at a 1∶1 (v/v) ratio with Laemmli sample buffer (#161-0737, Bio-Rad, Hercules, CA) with 350 mM DTT, brought to 95° for 8 min, and then centrifuged at 13,000 RPM for 10 min. Supernatants were used for the following analyses. *Hemoglobin and Myoglobin:* Proteins were separated from mixed muscle homogenates using SDS-PAGE, stained with Coomasie Brilliant Blue (Bio-Rad), and imaged using a ChemiDoc imaging system (Bio-Rad). Hemoglobin and myoglobin band densities were measured using ImageJ and calibrated using hemoglobin and myoglobin standards that were run simultaneously. *Western Blots:* Proteins were separated from gastrocnemius samples using SDS-PAGE and transferred to nitrocellulose membranes before blocking and immunoblotting for selected proteins. For complex I subunit NDUFB8 (MS105, Mitosciences, Eugene, OR), complex II subunit 30 kDa (MS203/C1217, Mitosciences), complex IV subunit IV (MS407, Mitosciences) and ANT1 (#9299, Santa Cruz Biotechnology, Santa Cruz, CA), membranes were blocked overnight at 4°C in 5% NFDM, primary antibodies were diluted 1∶1000 in 1% NFDM, and HRP-conjugated secondary antibodies (#7072, Cell Signaling, Danvers, MA) were diluted 1∶20000 in 1% NFDM. Actin (AB14128, Abcam, Cambridge, MA) was probed simultaneously as an internal loading control. For UCP3 (AB3477, Abcam), AMPK (#2532, Cell Signaling), and phospho-AMPK (#2535, Cell Signaling), membranes were blocked for 1 hour at room temperature in 3% BSA, primary antibodies were diluted 1∶1000 in 1% BSA, and HRP-conjugated secondary antibodies (#7074, Cell Signaling) were diluted 1∶2500 in 1% BSA. α-Tubulin (#2125, Cell Signaling) was probed simultaneously as an internal loading control.

### RT-PCR

RNA was extracted from liquid nitrogen pulverized gastrocnemius using the RNeasy Mini Kit (Qiagen, Valencia, CA) and cDNA libraries were created using the Omniscript RT Kit (Qiagen). Real time PCR was carried out using a Rotor-Gene 6000 (Corbett Research, Cambridge, UK). Primers were furnished by Dr. Rong Tian (UW Mitochondria and Metabolism Center, Seattle, WA) and analyses were performed using Rotor-Gene 6000 Series Software.

### F_2_-Isoprostanes

F_2_-isoprostanes were determined using a stable isotope dilution method with detection by gas chromatography/negative-ion chemical ionization/mass spectrometry (GC-NICI-MS) as previously described [51]. Briefly, 100–200 mg of mixed muscle powder was homogenized in ice-cold Folch solution (chloroform/methanol 2∶1) containing 5 mg/100 ml butylatedhydroxytoluene. Lipids were then extracted and chemically hydrolyzed with 15% KOH. After acidification with HCl, a stable isotope, 8-*iso*-prostaglandin F_2α_-d_4_ internal standard was added. Following extraction using C-18 and silica Sep-Pac cartridges, the eluted compounds were dried under nitrogen then converted to pentafluororobenzyl esters and purified by thin-layer chromatography. The purified F_2_-isoprostanes were derivatized to trimethysilyl ether derivatives then dissolved in undecane for quantification by GC/MS. Negative ion chemical ionization MS was performed by Agilent 6890 GC and Model 5975 MSD instruments with selected ions monitored for [^2^H_4_]15-F_2α_-IsoP internal standard (*m*/*z* 573) and F_2_-IsoPs (*m*/*z* 569).

### Calculations

A more detailed description of our calculations is available elsewhere [24]. A brief overview is presented below.

#### MR Spectroscopy

PCr/ATP ratios from fully relaxed spectra were multiplied by ATP concentrations from HPLC to find resting *in vivo* PCr concentration. Using this as a reference, the slope from Fit-to-Standard was converted to an absolute PCr concentration for each spectrum. PCr breakdown over time was fit using a least-squares linear approximation, and PCr recovery after cuff release was fit using a least-squares monoexponential approximation. These fits were used to calculate the resting ATP synthesis rate and maximum ATP synthesis rate [25], respectively. Intracellular pH and AMP concentrations were calculated using methods described elsewhere [52], [53].

#### Optical Spectroscopy

The oxygen saturation of myoglobin from PLS was multiplied by absolute myoglobin concentration to find moles of oxygen bound to myoglobin and used to calculate total dissolved oxygen in the intracellular compartment using known oxygen-myoglobin binding kinetics at 37°C [54]. An analogous approach was used to calculate moles of oxygen bound to hemoglobin and vascular dissolved oxygen using the oxygen saturation of hemoglobin from PLS. Oxygen consumption during ischemia was found using a least-squares linear approximation of change in total tissue oxygen content over time [24]. All measures of oxygen consumption were made before oxygen became limiting to respiration as previously described [55].

### Data Analysis

Statistical analyses were performed using Prism v5 (GraphPad, La Jolla, CA). In cases where three groups were compared (i.e. C, PQ1, and PQ2), one-way ANOVA was used to determine significance and Dunnett's post hoc test was used to determine significance of individual treatment groups vs. control. In cases where two groups were compared (i.e. PQ2 vs. C or SOD1^−/−^ vs. WT), two-tailed Student's t-tests were conducted. In figure 8C, two-way ANOVA was used to determine the effect of SOD1 knock-out on multiple electron transport chain complexes.

## Supporting Information

Figure S1 Heart rate (A) and arterial hemoglobin oxygen saturation (B) were measured before (0 days), after one week (7 days) and following two weeks of PQ treatment (14 days). There was no significant effect of PQ treatment on either variable at any time point. Data means±SEM, n = 5.
(TIF)

Figure S2 (A) A significant decrease in resting myoglobin (Mb) saturation with PQ treatment indicates an increased mitochondrial demand on oxygen delivery systems with uncoupling. n  = 8–9, * p<0.05. (B) There is a significant negative correlation between resting myoglobin saturation and *in vivo* mitochondrial oxygen consumption in PQ mice (p<0.0001).
(TIF)

Figure S3 Oxidative stress has the same effect on the *in vivo* metabolism of male mice as female mice. Treatment with 20 mg/kg PQ per week for two weeks leads to (A) decreased coupling of oxidative phosphorylation as measured by P/O ratio and (B) increased energy stress as measured by PCr/ATP ratio in male mice. Data means±SEM, n = 3, * p<0.05.
(TIF)

Figure S4 The absence of SOD1 leads to an increase in oxidative stress in skeletal muscle, as measured by F_2_-Isoprostanes in the gastrocnemius of female SOD1^−/−^ mice. Data means±SEM, n = 4, * p<0.05. Significance determined using one-tailed t-test.
(TIF)

Figure S5 Functional classification of genes differentially regulated in SOD1^−/−^ mice. Bars show the percentage of genes in the GO categories significantly overrepresented in the genes over expressed (A) or under expressed (B) in SOD1^−/−^ relative to WT in the EDL muscle using the GO FAT categories in NIH DAVID. Dark bars indicate categories associated with mitochondrial function. Array analysis used n = 4 per group. All categories listed are p<0.05.
(TIF)

Table S1 List of genes differentially expressed in the EDL of SOD1^−/−^ mice compared to WT. Genes are grouped into those uregulated and downregulated in SOD1^−/−^ and sorted by false discovery rate adjusted p-value (fdr_p). All genes are p<0.05.
(XLS)

Table S2 NIH DAVID Functional classification of genes listed in Table S1. GO categories containing more than five members with p<0.05 are listed. All categories, including redundant categories, are listed in the table. Data in the % column was used to generate Figure S5.
(XLS)

Methods S1 Detailed explanation of methods used to acquire data presented in Figures S1 and S5 and Tables S1 and S2.
(DOC)
