# Supplementary figures and images for: Reduced Coupling of Oxidative Phosphorylation In Vivo Precedes Electron Transport Chain Defects Due to Mild Oxidative Stress in Mice
Source: PLoS One. 2011 Nov 22;6(11):e26963. doi: 10.1371/journal.pone.0026963 (PMC3222658; doi:10.1371/journal.pone.0026963)

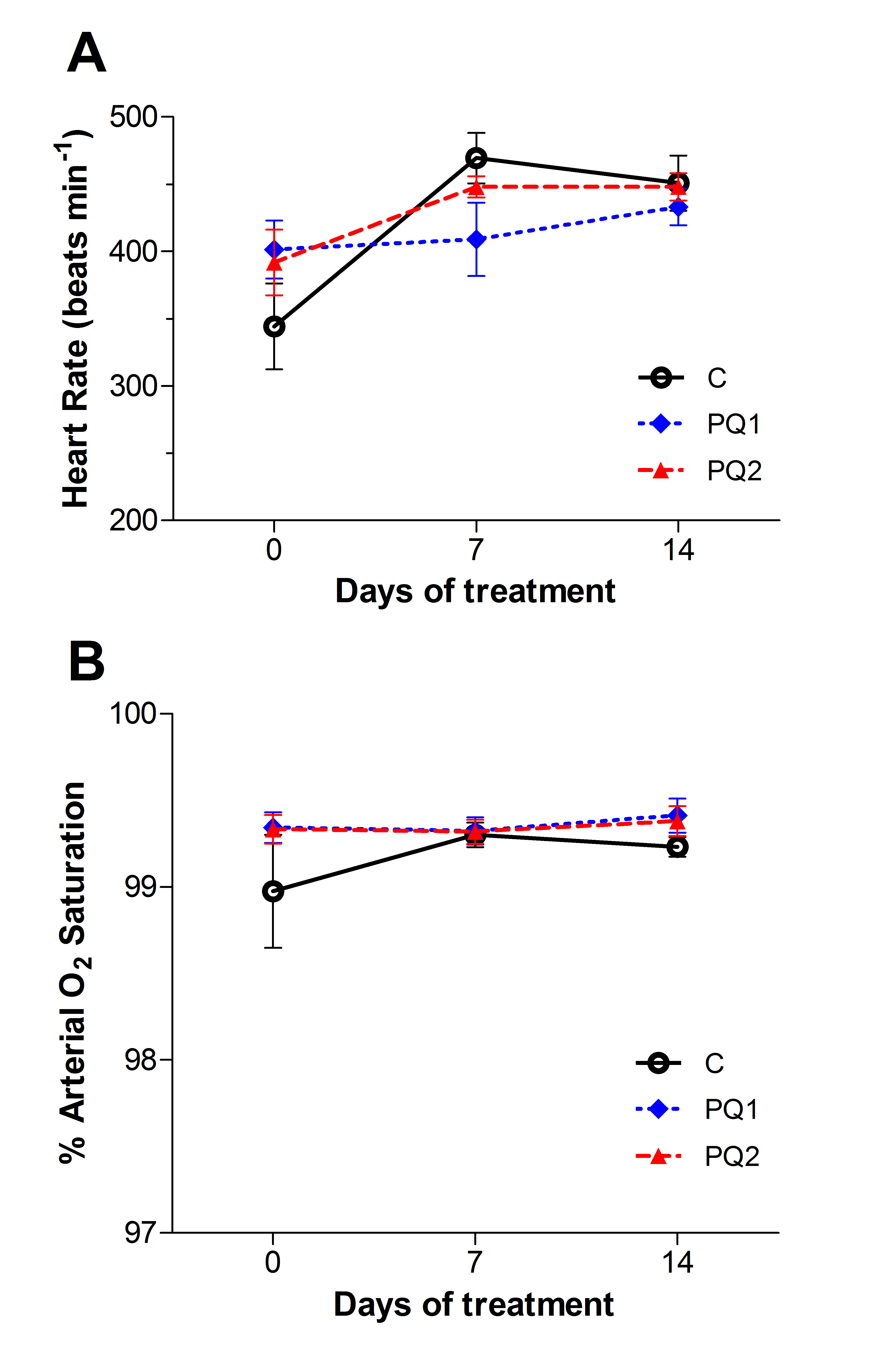

Supplement: Figure S1 — Heart rate (A) and arterial hemoglobin oxygen saturation (B) were measured before (0 days), after one week (7 days) and following two weeks of PQ treatment (14 days). There was no significant effect of PQ treatment on either variable at any time point. Data means±SEM, n = 5. (TIF) [file pone.0026963.s001.tif]

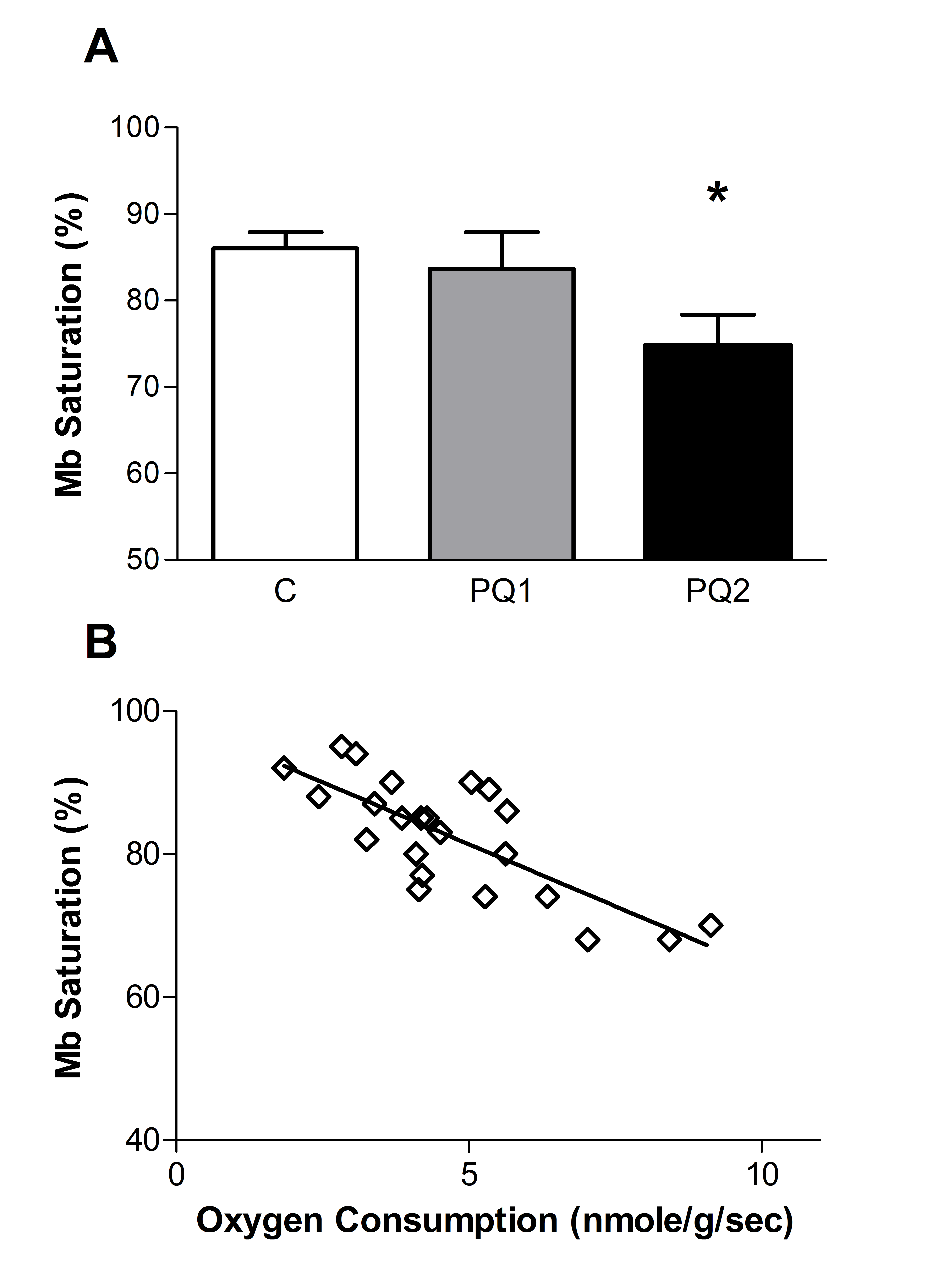

Supplement: Figure S2 — (A) A significant decrease in resting myoglobin (Mb) saturation with PQ treatment indicates an increased mitochondrial demand on oxygen delivery systems with uncoupling. n = 8–9, * p<0.05. (B) There is a significant negative correlation between resting myoglobin saturation and in vivo mitochondrial oxygen consumption in PQ mice (p<0.0001). (TIF) [file pone.0026963.s002.tif]

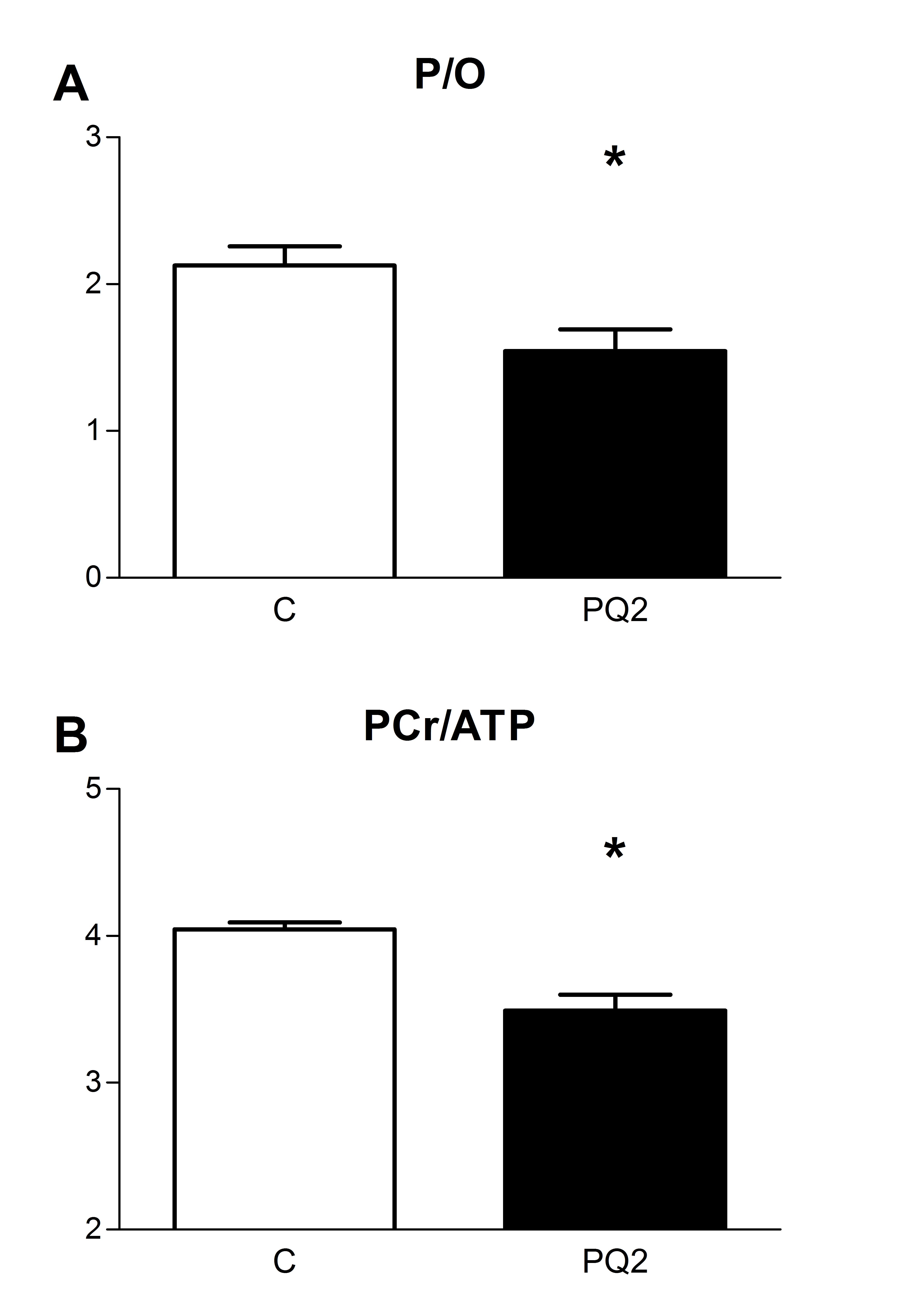

Supplement: Figure S3 — Oxidative stress has the same effect on the in vivo metabolism of male mice as female mice. Treatment with 20 mg/kg PQ per week for two weeks leads to (A) decreased coupling of oxidative phosphorylation as measured by P/O ratio and (B) increased energy stress as measured by PCr/ATP ratio in male mice. Data means±SEM, n = 3, * p<0.05. (TIF) [file pone.0026963.s003.tif]

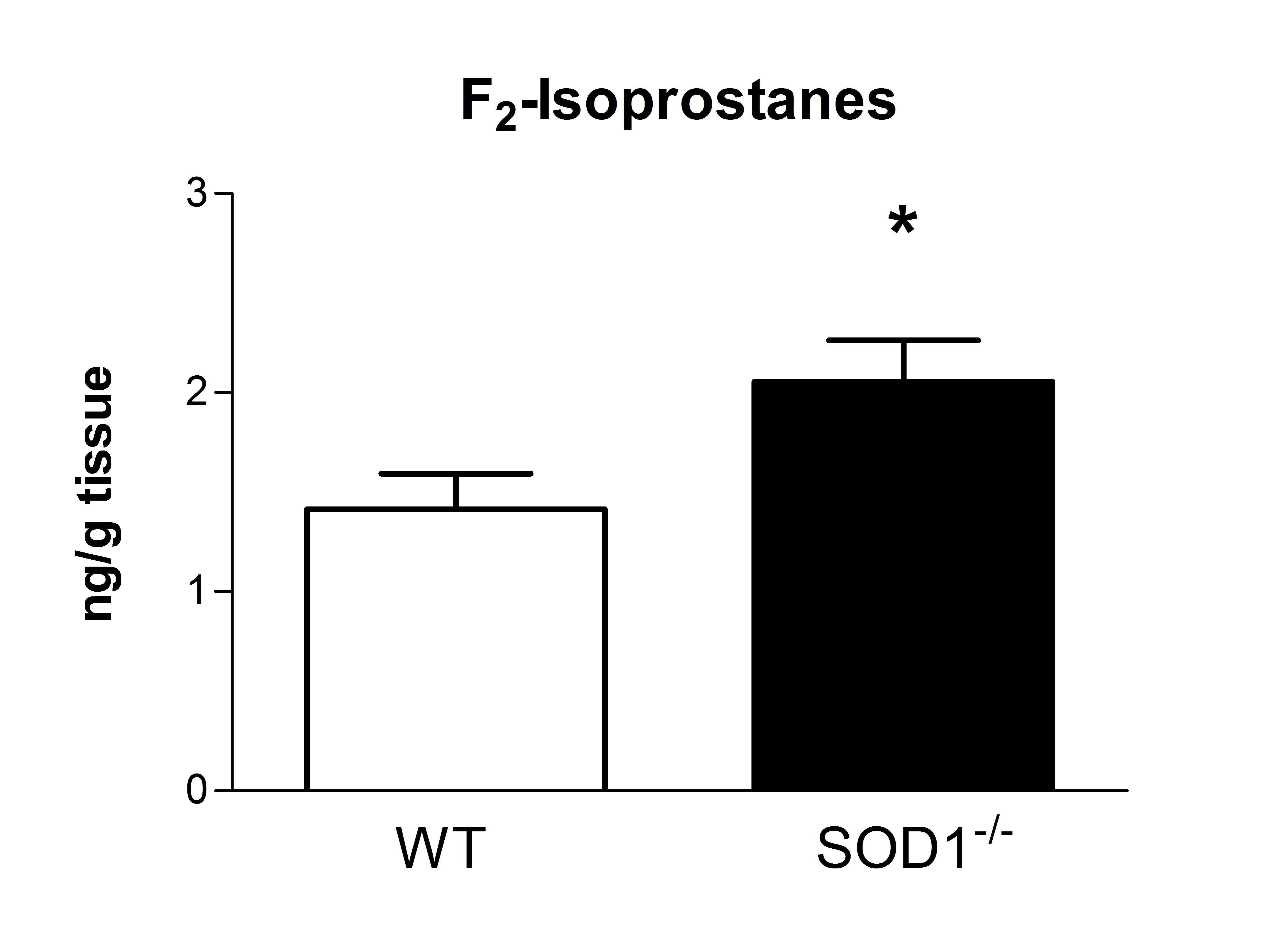

Supplement: Figure S4 — The absence of SOD1 leads to an increase in oxidative stress in skeletal muscle, as measured by F2-Isoprostanes in the gastrocnemius of female SOD1−/− mice. Data means±SEM, n = 4, * p<0.05. Significance determined using one-tailed t-test. (TIF) [file pone.0026963.s004.tif]

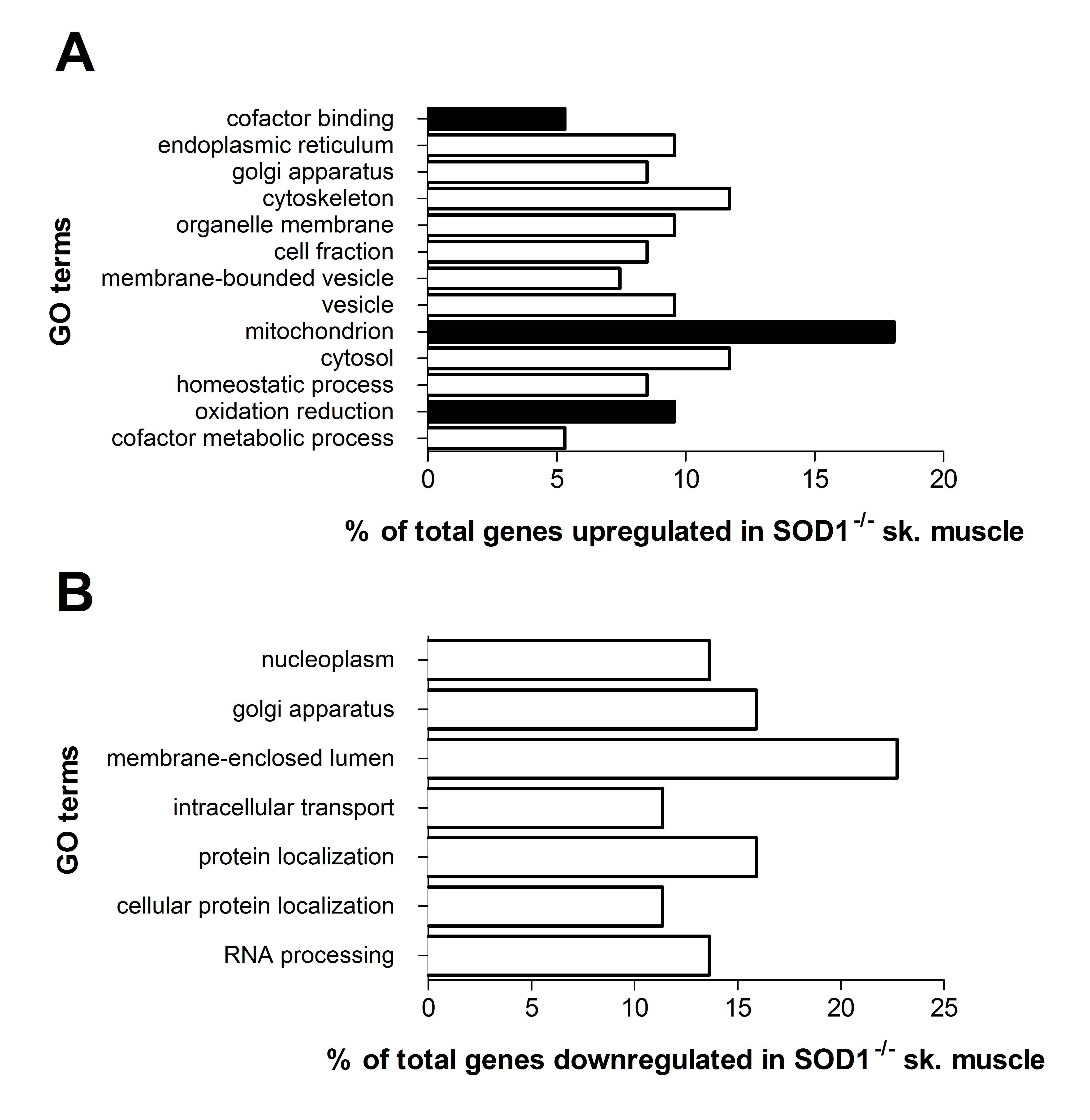

Supplement: Figure S5 — Functional classification of genes differentially regulated in SOD1−/− mice. Bars show the percentage of genes in the GO categories significantly overrepresented in the genes over expressed (A) or under expressed (B) in SOD1−/− relative to WT in the EDL muscle using the GO FAT categories in NIH DAVID. Dark bars indicate categories associated with mitochondrial function. Array analysis used n = 4 per group. All categories listed are p<0.05. (TIF) [file pone.0026963.s005.tif]
