# Supplementary material for: Reduced Coupling of Oxidative Phosphorylation In Vivo Precedes Electron Transport Chain Defects Due to Mild Oxidative Stress in Mice
Source: PLoS One. 2011 Nov 22;6(11):e26963. doi: 10.1371/journal.pone.0026963 (PMC3222658; doi:10.1371/journal.pone.0026963)
Supplement: Methods S1 — Detailed explanation of methods used to acquire data presented in Figures S1 and S5 and Tables S1 and S2. (DOC) [file pone.0026963.s008.doc]

**Supplemental Methods**

*Pulse Oximetry*

Resting heart rate and arterial oxygen saturation were measured longitudinally in anesthetized mice using the MouseOx® pulse oximeter (Braintree Scientific, Inc., Braintree, MA) 0, 7, and 14 days into PQ treatment. Mice were anesthetized using a gas mixture of 2% isoflurane, 98% oxygen, hair was removed from a small area of the chest and neck using over-the-counter hair removal cream (Nair, Church & Dwight Co., Princeton, NJ), and an infrared sensor was clipped to the skin over the carotid artery to make measurements.

*DNA Microarray*

All microarray data is MIAME compliant and has been deposited in the Gene Expression Omnibus (GEO) database under accession number GSE28837, sample numbers GSM714165 through GSM714172.

Total RNA from EDL muscle was isolated using the RNeasy Fibrous Tissue Mini Kit (QIAGEN, Valencia, CA). The quantity of the RNA was determined by Nanodrop (Thermo Scientific, Wilmington, DE) and the RNA integrity number (RIN) was confirmed with the Agilent 2100 Bioanalyzer (Agilent Technologies, Palo Alto, CA).

Microarray data was acquired using the Sentrix MouseRef-8 V1.1 Expression Beadchip (Illumina, San Diego, CA) and normalized with Illumina BeadStudio data analysis software. The raw gene intensity was initially transformed to a logarithmic (base 2) scale and then quantile normalization was applied to remove the systematic variation between microarray chips [1]. The normalized data was processed and analyzed with SAS (V9.13).

Genes differentially regulated in SOD1^-/-^ mice were identified using liberal criteria (false discovery rate corrected p-value<0.05 and 1.2 fold change in expression) to investigate trends in the response to the absence of SOD1. We then used the NIH Database for Annotation, Visualization and Integrated Discovery (DAVID) software [2,3] to identify functional categories of genes up and down regulated in the SOD1^-/-^ mice. We used the default GO FAT categories for Biological Processes, Cellular Constituent, and Molecular Function groupings to minimize the redundant categories identified in the DAVID Functional Annotation Chart analysis. Categories with p<0.05 and gene count number greater than 5 were deemed significant.

References

1. Bolstad BM, Irizarry RA, Anstrand M, Speed TP (2003) A comparison of normalization methods for high density oligonucleotide array data based on variance and bias. Bioinformatics 19: 185-193.

2. Huang DW, Sherman BT, Lempicki RA (2009) Systematic and integrative analysis of large gene lists using DAVID Bioinformatics Resources. Nature Protocols 4: 44-57.

3. Dennis GJ, Sherman BT, Hosack DA, Yang J, Gao W, et al. (2003) Database for Annotation, Visualization, and Integrated Discovery. Genome Biology 4: P3.
